# Supplementary material for: Content-rich biological network constructed by mining PubMed abstracts
Source: BMC Bioinformatics. 2004 Oct 8;5:147. doi: 10.1186/1471-2105-5-147 (PMC528731; doi:10.1186/1471-2105-5-147)
Supplement: Additional File 2 — The original results of the above study (non-essential files are deleted to keep the file size under the limit set by BMC bioinformatics). [file 1471-2105-5-147-S2.bz2 › chilibotAdditionalFile2/dip10/101ID7937747E430/html/BCL2L1_BCL2.html]

 


 **BCL2L1** and **BCL2** 
  
Found 1846 abstracts in PubMed, retrieved 10.  
 

 What does Google say? 
 PDF only 
| .edu only 

---

**Interactive relationship** (e.g. stimulation, inhibition, etc)

**Inhibitory relationship**- In addition, detailed knowledge of the structures of Bcl 2  [ **BCL2** ]  and Bcl X  [ **BCL2L1** ]  L, coupled with high throughput and computer assisted screening of chemical libraries, has led to the identification of a number of short peptides and small organic molecules capable of inhibiting Bcl 2  [ **BCL2** ]  and Bcl X  [ **BCL2L1** ]  L function.  Ref: 12886234 Leukemia, 2003
**Stimulatory relationship**- Multiplex RT PCR of apoptosis pathway related genes revealed that Bcl 2  [ **BCL2** ]  is absent and Bcl xS  [ **BCL2L1** ]  message increases in FHIT transfected clones.  Ref: 12890991 Pancreatology, 2003
**Neutral relationship**

**Non-interactive relationship** (e.g. studied together, co-existance, homology, etc.)

- AIMS To correlate the expression of a series of apoptotic and oncogene markers including p53, Bcl 2  [ **BCL2** ] , BAX, Bcl XL  [ **BCL2L1** ] , p21WAF, 1 CIP1, cyclin D1, HER 2 neu in thymic epithelial tumours with histological type, stage and resectability and to determine whether the information on HER 2 neu would be valuable in identifying patients who are eligible for anti HER 2 neu treatment.  Ref: 12877732 Histopathology, 2003
- As Bcl 2  [ **BCL2** ]  and Bcl X  [ **BCL2L1** ]  are co expressed in EBV transformed B lymphocytes, we attempted to determine their status in malignant NPC cells.  Ref: 12893071 Cancer Detect Prev, 2003
- In the present study, we have examined the roles played by the Bcl 2  [ **BCL2** ]  family members Bcl xL  [ **BCL2L1** ]  and Bid in regulating this response.  Ref: 12893848 J Pharmacol Exp TherJ Pharmacol Exp Ther, 2003
- Furthermore, Bcl 2  [ **BCL2** ]  family members Bad, Bak, and Bcl xS  [ **BCL2L1** ]  protein levels were increased in FHIT transfected clones when compared with Panc 1 cells.  Ref: 12890991 Pancreatology, 2003
- Bcl X  [ **BCL2L1** ]  and Bcl 2  [ **BCL2** ]  are co expressed in 89% of NPCs whereas their expression is mutually exclusive in other head and neck carcinomas particularly squamous cell carcinomas, SCC.  Ref: 12893071 Cancer Detect Prev, 2003
- ... other Bcl 2  [ **BCL2** ]  family proteins including Bax, Bcl 2  [ **BCL2** ] , Bcl XL  [ **BCL2L1** ] , and Bag remained unchanged.  Ref: 12874837 J Cell Biochem, 2003
- We demonstrated that Dox and Taxol induced apoptosis through down regulation of XIAP and phosphorylation of Bcl 2  [ **BCL2** ]  in a concentration dependent manner without changing expression of Bcl xL  [ **BCL2L1** ]  in H460 cells.  Ref: 12883037 Mol Cancer Ther, 2003
- Similar BCL X  [ **BCL2L1** ]  but different BCL 2  [ **BCL2** ]  levels in the two age groups of north African nasopharyngeal carcinomas.  Ref: 12893071 Cancer Detect Prev, 2003
- The defective apoptosis seen in human cancers often results from overexpression of antiapoptotic proteins in the Bcl 2  [ **BCL2** ]  protein family, particularly Bcl 2  [ **BCL2** ]  and Bcl X  [ **BCL2L1** ]  L.  Ref: 12886234 Leukemia, 2003
- These newly described agents hold considerable promise for enhancing the chemo and radiation sensitivities of Bcl 2  [ **BCL2** ]  and Bcl X  [ **BCL2L1** ]  L overexpressing cancers.  Ref: 12886234 Leukemia, 2003
- There was no difference found in the expression of proapoptotic Bax and Bcl X  [ **BCL2L1** ]  s or antiapoptotic Bcl 2  [ **BCL2** ]  and Bcl X  [ **BCL2L1** ]  L genes nor in the expression of the tumor suppressor gene p53.  Ref: 12885947 J Leukoc Biol, 2003
- Selegiline and its metabolite, desmethylselegiline, reduce apoptosis by altering the expression of a number of genes, for instance, superoxide dismutase, Bcl 2  [ **BCL2** ] , Bcl xl  [ **BCL2L1** ] , NOS, c Jun, and nicotinamide adenine nucleotide dehydrogenase.  Ref: 12880486 Antioxid Redox Signal, 2003
- The apoptotic pathways thought to be employed by epidermal keratinocytes in the various cell layers are reviewed, with special emphasis on the protective mechanisms such as proto oncogenes bcl 2  [ **BCL2** ]  and Bcl XL  [ **BCL2L1** ] , growth factors, and the NFkB pathway in protecting keratinocytes from premature apoptosis during the process of upward migration and differentiation.  Ref: 12884460 J Drugs Dermatol, 2003
- Bcl 2  [ **BCL2** ]  and Bcl X  [ **BCL2L1** ]  expression was assessed semi quantitatively using a score based on the percentage of positive cells and staining intensity.  Ref: 12893071 Cancer Detect Prev, 2003
